# Eating behaviour in Swiss preschool children–Validation of a German and a French version of the Children’s Eating Behaviour Questionnaire (CEBQ)

**DOI:** 10.1371/journal.pone.0295259

**Published:** 2023-12-07

**Authors:** Anaëlle L. Leuba, Andrea H. Meyer, Tanja H. Kakebeeke, Kerstin Stülb, Amar Arhab, Annina E. Zysset, Claudia S. Leeger-Aschmann, Einat A. Schmutz, Susi Kriemler, Oskar G. Jenni, Jardena J. Puder, Simone Munsch, Nadine Messerli-Bürgy

**Affiliations:** 1 Department of Psychology, University of Fribourg, Fribourg, Switzerland; 2 Institute of Psychology, University of Lausanne, Lausanne, Switzerland; 3 Department of Clinical Psychology and Psychotherapy, University of Fribourg, Fribourg, Switzerland; 4 Department for Psychology, University of Basel, Basel, Switzerland; 5 Child Development Center, University Children’s Hospital Zurich, Zurich, Switzerland; 6 Children’s Research Center, University Children’s Hospital Zurich, Zurich, Switzerland; 7 Obstetric Service, Department Women-Mother-Child, Lausanne University Hospital, Lausanne, Switzerland; 8 Epidemiology, Biostatistics and Prevention Institute, University of Zurich, Zurich, Switzerland; St John’s University, UNITED STATES

## Abstract

Young children’s eating behavior is crucial for any further development of healthy eating. Early eating behavior are often assessed through parental report. The Children’s Eating Behaviour Questionnaire (CEBQ) is a widely used parental questionnaire that has been validated in families of different gender, age and cultural background. Research has shown that the 8-factor structure has some inconsistencies and sample characteristics such as age, gender, and culture can influence the results. To which extent such sample characteristics might influence results within a multi-lingual culture has not been investigated so far. Therefore, the aim of the study was to evaluate the factor structure of the CEBQ among 511 preschool children of the French and German parts of Switzerland, aged 2 to 6 years (Mean 3.85 years; SD 0.69). Confirmatory Factor Analysis showed a modified structure of the original questionnaire, with a 7-factor structure providing a reasonable fit to the data (TLI = 0.954, CFI = 0.952, RMSEA = 0.063 and SRMR = 0.067). The subscale ‘Desire to drink’ was removed, and a few items moved to other subscales as they loaded higher on a different subscale compared to the original model. Reliabilities based on the coefficient omega were acceptable to satisfying across the seven factors, ranging from 0.66 to 0.90. There were no significant gender or age differences, but French speaking children showed higher levels of ‘Satiety responsiveness’ and lower ‘Enjoyment of food’ than German speaking children. Yet, these effects were small. The German and French CEBQ are valid and reliable versions of the original CEBQ and can be used in a multicultural context.

## Introduction

Problems of eating behaviour, such as altered speed in eating or response to satiety, at an early age is a worldwide issue. It can put individuals at risk of developing eating disorders and other diseases in later childhood and in teenage years, thereby having a long-term impact on mental and physical health in human beings as not only physical, but also cognitive and emotional development is ongoing and crucial at this age [1–6]. The onset of eating behaviour problems is believed to set at a young age [2] and is linked to an increased risk for unhealthy development that leads to obesity or other eating disorders at a later age [7] including difficulties related to body image, self-esteem and the accomplishment of age-related developmental tasks such as autonomy and identity development. Differences in eating behaviour can also already be found in premature babies and babies with feeding problems show less enjoyment of food, less appetite, more slowness in eating and a higher satiety responsiveness [8] than full-term babies or babies without any feeding problems. Main features of eating behaviour (e.g. food approach or food avoidance resulting in picky eating or loss of control eating) seem to be at a starting point at preschool age and show a continuity throughout childhood [1, 9]. Further, eating patterns at an early age can be connected to other symptoms of self-regulation deficits and might therefore represent early signs of problematic emotion and impulse regulation patterns. Dysfunctional eating patterns are known to be connected to attention and hyperactivity problems [10], with difficulties in inhibitory control as underlying mechanisms [11]. Food avoidance or picky eating are part of eating patterns seen in children with problems within the autism-spectrum [12]. While an early assessment of such behavior patterns lays the ground for tailored prevention or intervention, we would like to underline that so far there is no evidence on causal relationships between such early eating patterns and autism-spectrum disorder. Therefore, understanding the eating behaviour at this early period of life might help to intervene at an early point of development of eating behaviour problems [3] and prevent further problematic developments.

The Children’s Eating Behaviour Questionnaire (CEBQ), developed and validated in the UK by Wardle and colleagues [13] is a psychometric instrument to assess eating behaviour in young children (between 2 and 9 years) via parental report. The questionnaire was created to alleviate the issues a laboratory assessment can cause. It is as efficient in assessing children’s eating behaviour as behavioural tests [14]. The CEBQ has been developed by merging results of interviews with parents and by modifying questions of already existent scales that focus on parental assessments of the child’s eating behaviour [13]. The underlying idea was to confirm the parent’s understanding of theoretically developed constructs covering different dimensions of eating behavior. It consists of eight different subscales of eating behaviour. The subscales include the following aspects: ‘Food Responsiveness’ (FR) measuring the responsiveness to external cues of food such as the smell or sight of food [2]; ‘Enjoyment of Food’ (EF) evaluating the pleasure of eating with or without hunger; ‘Emotional Overeating’ (EOE) measuring the tendency to eat under the pressure of emotions; ‘Desire to Drink’ (DD) measuring the desire of the child to have drinks with him and/or the want of sweetened drinks; ‘Satiety Responsiveness’ (SR) measuring the responsiveness to internal satiety signals; ‘Slowness in Eating’ (SE) evaluating the rate of speed during an entire meal, ‘Emotional Undereating’ (EUE) examining the tendency to eat less when under pressure of emotions, and ‘Food Fussiness’ (FF) that evaluates the attitude towards food choices. These eight subscales of the CEBQ have been referred to the two dimensions ‘Food Approach’ and ‘Food Avoidance’ [15–20]. Food Approach comprises the four subscales EF, EOE, DD and FR, while Food Avoidance comprises the four subscales SR, SE, EUE and FF. The two dimensions have been associated with weight in preschool age. Higher score in Food Approach subscales has been related to increased weight and higher score in Food Avoidance subscales to lower weight conditions [3, 18, 20]. Indeed, Food Approach and Food Avoidance are often set as opposite dimensions referring to the eight subscales, however, only one study investigated whether the theoretical distinction between the two dimensions could be empirically supported, with mixed evidence [15]. Furthermore, several studies investigated the original 8-factor structure of the original CEBQ by Wardle et al. [13], some of which confirmed the original 8-factor structure [15, 16, 22], while several others obtained a 7-factor structure [3, 18–22], and one a 6-factor structure [17].

These inconsistent results regarding the factor structure have been discussed to be influenced by sample characteristics. For instance, different factor structures were found in samples considering different cultural populations within English speaking countries (e.g., different ethnic groups) [16, 22], but also in non-English speaking European [18, 23, 24] and Asian countries [25–27] which used translated versions of the CEBQ. Further, age has been discussed to influence the factor structure of the CEBQ as repeated assessment over a one-year period in a multi-ethnic sample of 3 years olds resulted in different findings [28]. Besides this, food avoidant behaviour tends to decrease, whereas food approaching behaviour rather increases over time in children from 4 to 10 years [1] due to changes of the child’s food environment which includes an increase in food choices at an older age and a loss of monitoring of parents [1, 2, 29]. In addition, gender differences of eating behaviour have been discussed although controversially over different age periods. Whereas higher FF can be found in girls than in boys in toddlers [25], the opposite can be seen at the age of 6 and 7 [18]. Furthermore, boys showed more EOE than girls in a Dutch sample but less EF at preschool age in the same sample [18], whereas boys aged 6 to 11 years living in Thailand showed more EF than girls [27]. However, boys were more food responsive in toddlerhood [28], and showed less food avoidant behaviour at the age of 6 and 8 years [30]. In addition, all these studies considered different methodological approaches which might have added to the variation of the results in different studies. Thus, evidence on the influence of sample characteristics on eating behaviour assessment is not consistent and could potentially impact the assessment in children within a preschool age range and differ from those at preschool age.

To our knowledge, the CEBQ has not been translated and used in a French and German sample. Furthermore, no studies investigated the potential difference in a multilingual country. To sum up, there is no clear evidence for a consistent factor structure of a French and German translation of the CEBQ so far and it remains unclear, whether sample characteristics (e.g., gender, age and language area) might have an impact on a multilingual sample. Therefore, the aim of the study was (a) to validate the original factor structure of a French and German translated version of the CEBQ in a Swiss preschool community sample and (b) to identify the impact of sample characteristics as expressed by age, gender and language area on the different factors obtained.

## Method

### Study sample and design

The Swiss Preschooler’s Health Study (SPLASHY) is a multi-site prospective cohort study including 555 children during early childhood within two sociocultural areas of Switzerland (German and French speaking part) (ISRCTN41045021). SPLASHY aimed at understanding the impact of stress and physical activity on the development of weight problems and behavioral problems (for details [31]). Children were recruited from 84 childcare centers within five cantons of Switzerland (Aargau, Bern, Fribourg, Vaud, Zurich). These five cantons together made up 50% of the Swiss population in 2013. To attain a large external validity, all children aged 2 to 6 years were included. Recruitment started between November 2013 and October 2014. The detailed study design and the overall objectives have been previously described [30]. The study was approved by all local ethical committees (No 338/13 for the Ethical Committee of the Canton of Vaud as the main ethical committee) and is in accordance with the Declaration of Helsinki. Parents provided written informed consent. The current analysis focuses on the baseline cross-sectional data collected between February 2014 and November 2015. After parents’ written consent, all parents received a link to an online set of questionnaires to complete.

### Assessment

*Eating behaviour* was assessed by the Children’s Eating Behaviour Questionnaire (CEBQ) [13] which includes eight subscales and 35 items and is known to be a valid and reliable parental assessment tool for children aged 2 to 9 years [13, 14]. For this study, the original version by Wardle et al. [13] was translated into a German and a French version by German and French native speakers who were all fluent speakers of the English language and all working at the bilingual (French and German speaking) University of Fribourg, Switzerland. Translation of the questionnaires integrated forward and backward translations by both experts in the field of eating behavior and regional language use (JJP, SM) until inconsistencies could be removed.

In the final CEBQ, parents were asked to respond to different questions on the eating behaviour of their child by using a 5 point-Likert scale ranging from “never” (1) up to “always” (5) as in the original version of Carnell & Wardle [14]. The questionnaire includes eight subscales of eating behaviour: Food responsiveness with five items (e.g. “given the choice, my child would eat most of the time”), Enjoyment of food with four items (e.g. “my child enjoys eating”), Emotional overeating with four items (e.g. “my child eats more when worried), Desire to drink with three items (e.g. “if given the chance, my child would drink continuously throughout the day”), Satiety responsiveness with five items (e.g. “my child gets full before his/her meal is finished”), Slowness in eating with four items (e.g. “my child eats slowly”), Emotional undereating with four items (e.g. My child eats less when upset), and Food fussiness with six items (e.g. “my child refuses new food at first”).

*Age of the child* was assessed by calculation of the exact age at the time of assessment using the birth date and the assessment time point at baseline. Further, parents were asked to provide the *gender of their child* and their occupational status which was transformed into an ISEI value (International Socio-Economic Index) [32]. Scores range between 16 and 90. Lowest value represents an unskilled worker or cleaner and highest value a judge. Occupational status of both parents was assessed and coded. The maximum socio-economic status (SES) of both parents was used as the *SES level of the child*. *Language area* was defined by the language of the childcare center of each child living either in the French or the German part of Switzerland.

### Statistical analysis

Two confirmatory factor analysis (CFA) models were set up, one based on the original model by Wardle and colleagues [13], the second based on a modified version, including only 7 of the 8 original factors. As all items were measured on a Likert scale with five levels and were hence ordinally scaled, the mean and covariance adjusted weighted least squares estimator (WLSMV) was used to compute parameters and their standard errors. To report model fit indices, the respective robust variants including the comparative fit index (CFI), the Tucker Lewis index (TLI), the root mean square error of approximation (RMSEA), and the standardized root mean square residual (SRMR) are provided. Acceptable model fit requires the following criteria for these indices: RMSEA (≤ 0.06, 90% CI ≤ 0.06, CFit not significant), SRMR (≤ 0.08), CFI (≥ 0.95), and TLI (≥ 0.95) [33]. All analyses were performed using the software R (R Core Team, 2020), including the R package lavaan [34].

A multiple-indicators and multiple-causes (MIMIC) model [35] was used to assess differential item functioning, i.e. assessing the influence of children’s gender, age, and Swiss language area (German or French) as explanatory variables on the means of the seven factors as defined in the modified CFA model. Descriptive statistics were calculated using means and standard deviations, or percentages for categorical data. To estimate reliabilities of the factors obtained, we used the omega coefficient [36] and the Cronbach’s alpha as the latter has often been criticized [37].

## Results

### Descriptive statistics

In total, we collected the parents’ reports of 555 children whereof data of 511 children could be kept in the analyses (parents of 44 children showed incomplete responding to the CEBQ and therefore these questionnaires had to be excluded from the analysis). Mean age of the children was 3.85 years (SD = 0.69), and 47% were girls. A total of 76% were living in the German-speaking part of Switzerland and completed the German version of the questionnaire and 24% were living in the French-speaking part and completed the French version. Mean SES was 62.88 (SD = 14.97) and slightly higher than in the Pisa study (Swiss sample = 53.00) of OECD (Organization for Economic Co-operation and Development) countries [38].

### Factorial validity and internal reliability of the CEBQ

**Fig 1:**
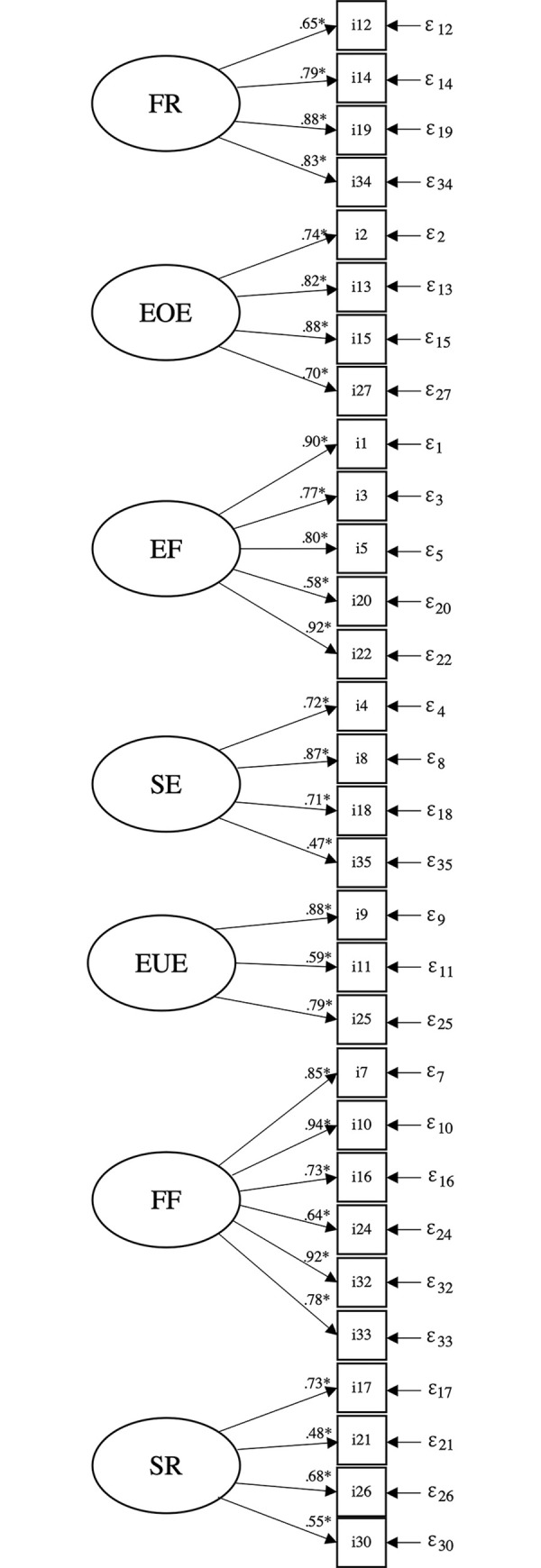
The 7-factor structure of the Children’s Eating Behaviour Questionnaire.

The original 8-factor structure as suggested by Wardle and colleagues [13] led to a poor model fit in the presented study (TLI = 0.920, CFI = 0.929, RMSEA = 0.069 and SRMR = 0.081). Our modified CFA model variant presented in Fig 1 had a clearly improved model fit which was satisfactory (TLI = 0.952, CFI = 0.957, RMSEA = 0.061 and SRMR = 0.068) and contained the following modifications: First, the factor DD (consisting of three items, of which item 29 “if given the chance, my child would drink continuously throughout the day” had a standardized loading above 1 and a negative error variance), was dropped, leading to a 7-factor model. Second, two other items for which modification indices reported high loadings on several other factors in both cases (item 23 “My child eats more when s/he is happy” and item 28 “Even if my child is full up, s/he finds room to eat his/her favorite food”) had to be removed. Third, item 3 in the original questionnaire (“My child has a big appetite”) needed to be transferred from its original factor SR to the factor EF, as it loaded much higher on the latter.

The reliabilities of the seven factors were in the range between 0.66 (SR) to 0.90 (FF) at baseline (see Table 1) and therefore comparable to the original version showing internal consistencies between 0.72 to 0.91 [13].

**Table 1:** Descriptive statistics and reliabilities based on the omega coefficient for each factor.

CEBQ Factors	M (SD)	Omega/ Cronbach alpha coefficient
Food Responsiveness, 4 items	2,2 (0,73)	0,83 / 0,79
Emotional Overeating, 4 items	1,5 (0,56)	0,77 /, 0,75
Enjoyment of Food, 5 items	3,8 (0,69)	0,86 / 0,84
Satiety Responsiveness, 4 items	2,8 (0,61)	0,66 / 0,69
Slowness in Eating, 4 items	2,9 (0,75)	0,76 / 0,72
Emotional Undereating, 3 items	2,9 (0,84)	0,78 / 0,75
Food Fussiness, 6 items	2,9 (0,8)	0,90 / 0,89

*Note*. Behaviours are rated on a five-point Likert scale. These are the factors retained for our 7-factors structure model.

Fig 1 shows the loadings of our proposed 7-factor model based on confirmatory factor analysis of the Children’s Eating Behaviour Questionnaire (CEBQ) for our proposed 7-factor model. Food Responsiveness (FR), Emotional Overeating (EOE), Enjoyment of Food (EF), Slowness in Eating (SE), Emotional Undereating (EUE), Food Fussiness (FF), and Satiety Responsiveness (SR). “i” means items, “ε” means error.

Correlations among the seven factors were particularly high between FR and EOE (*r* = .75), between EF and SR (*r* = –.58), and between FF and EF (*r* = –.60) (Table 2).

**Table 2:** Correlations for factors.

Subscales	1	2	3	4	5	6	7
1. Food Responsiveness (FR)	-						
2. Emotional Overeating (EOE)	**.75** **	-					
3. Enjoyment of Food (EF)	**.49** **	**.11** *	-				
4. Satiety Responsiveness (SR)	**–.26** **	–.06	–.58**	-			
5. Slowness in Eating (SE)	**-.13** **	–.01	–.40**	**.34** **	-		
6. Emotional Undereating (EUE)	-.03	**.20** **	**–.34** **	**.46** **	**.24** **	-	
7. Food Fussiness (FF)	**–.13** **	.04	**-.60** **	**.34** **	**.19** **	**.32** **	-

*Note*. Factors of our proposed 7-factor structure model.

*p < .05.

** *p* < .01.

The higher-order model, including the two scales Food Approach and Food Avoidance [14] could not be supported in this study. The higher order model based on the originally proposed 8-factor solution led to a poor model fit (TLI = 0.884, CFI = 0.894, RMSEA = 0.095 and SRMR = 0.110), with the two higher-order factors correlating highly negatively with each other (*r* = –.76). Closer inspection of the model revealed that the inclusion of Food Avoidance was reasonable, but not so for Food Approach and we therefore did not consider higher-order models in further analyses.

### Influence of gender, age, and language area on the CEBQ subscales

**Fig 2:**
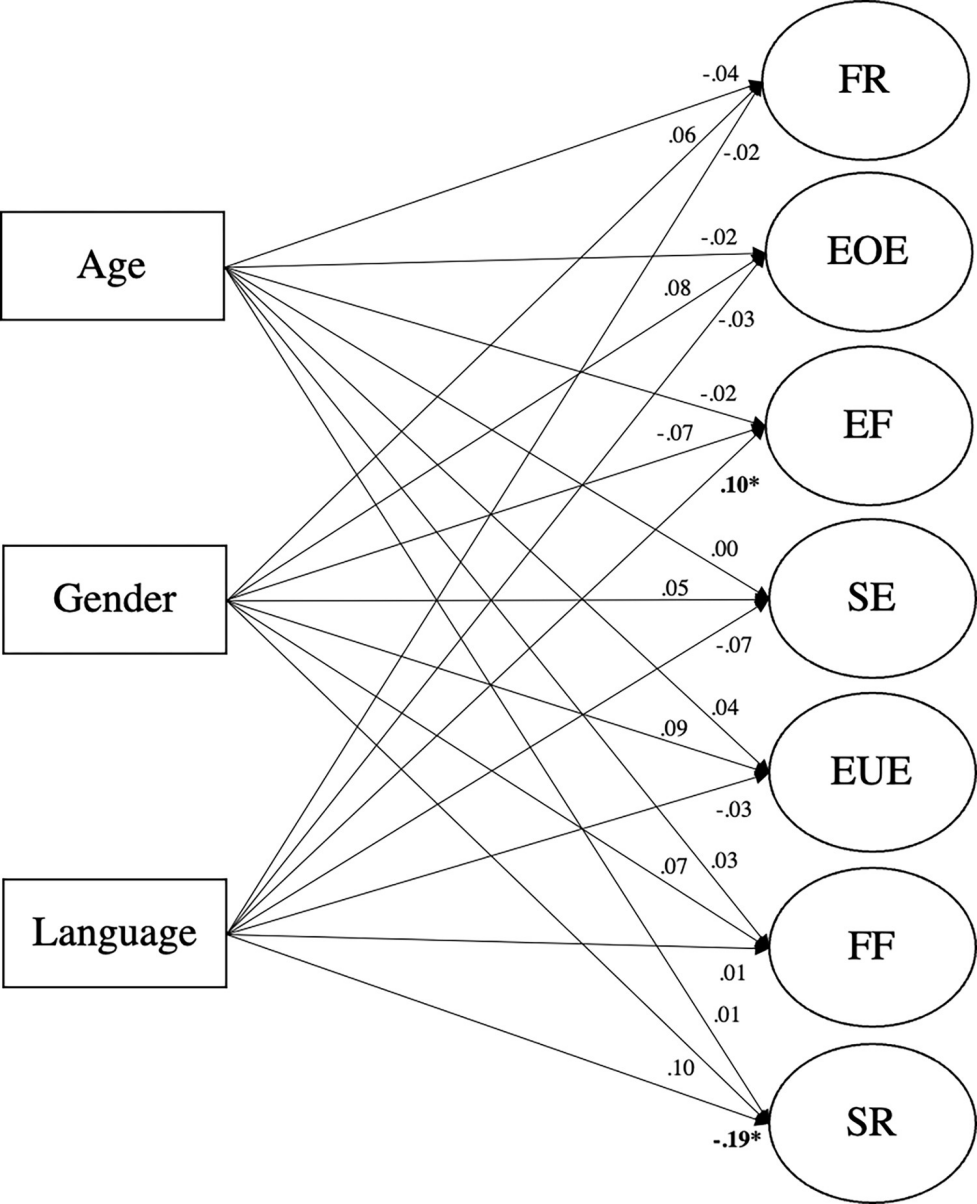
MIMIC model of our proposed 7-factor structure of the CEBQ. A MIMIC model based on our proposed 7-factor model of the Children’s Eating Behaviour Questionnaire. Explanatory variables were age, gender, and language (French and German) in a Swiss population of children between the age of 2 to 6 years old. Numbers denote the standardized regression coefficients. * *p* < 0.05.

Fig 2 shows the results from the MIMIC model with the seven factors regressed on the three variables age, gender, and language area. The fit of this model was satisfactory (TLI = 0.954, CFI = 0.952, RMSEA = 0.063 and SRMR = 0.067). The correlations among the three variables were very low, ranging between .014 and .081, and were set to 0 in the MIMIC model. There was no influence of age and gender on the loadings of the factors. Only for language area did we find an influence on two factors: satiety responsiveness an enjoyment of food (see Table 3). Thus, French speaking children showed higher values for satiety responsiveness than their German speaking counterparts, the effect size being small to medium (standardized path coefficient = -0.19). A closer inspection of this factor revealed that the values for three out of the four items of this factor (“… gets full before meal is finished”, “… gets full up easily”, “… cannot eat a meal if had a snack just before”) were increased in the French relative to the German speaking children. The fourth item “… leaves food on plate at the end of a meal” of this subscale did not differ between language areas. In addition, the French speaking children showed lower values in enjoyment of food, the effect size being small (standardized path coefficient = 0.11).

**Table 3:** Regression coefficients of the influence of explanatory variables age, gender, and language area on the seven factors of the CEBQ.

Effect	Estimate	*SE*	z-value^1^	*p-*value	Standardized Estimate
Food Responsiveness ~					
Age	-0.038	0.048	-0.793	0.428	-0.040
Gender	0.076	0.066	1.145	0.252	0.058
Language	-0.028	0.076	-0.368	0.713	-0.018
Emotional Overeating ~					
Age	-0.026	0.059	-0.446	0.656	-0.024
Gender	0.115	0.081	1.409	0.159	0.077
Language	-0.061	0.094	-0.644	0.520	-0.035
Enjoyment of Food ~					
Age	-0.028	0.055	-0.514	0.607	-0.024
Gender	-0.116	0.077	-1.512	0.131	-0.072
Language	0.197	0.087	2.273	0.023	**0.105** *
Satiety Responsiveness ~					
Age	0.014	0.058	0.234	0.815	0.012
Gender	0.152	0.080	1.884	0.060	0.100
Language	-0.333	0.091	-3.673	0.000	**-0.187** **
Slowness in Eating ~					
Age	0.004	0.050	0.082	0.935	0.004
Gender	0.079	0.073	1.085	0.278	0.054
Language	-0.126	0.088	-1.431	0.152	-0.073
Emotional Undereating ~					
Age	0.047	0.064	0.731	0.465	0.037
Gender	0.159	0.088	1.800	0.072	0.091
Language	-0.054	0.105	-0.513	0.608	-0.026
Food Fussiness ~					
Age	0.037	0.056	0.665	0.506	0.030
Gender	0.112	0.079	1.429	0.153	0.066
Language	0.015	0.090	0.170	0.865	0.008
					

*Note*. Gender is coded as Males = 1, Females = 2. Language area is coded as French speaking = 1, German speaking = 2.

*p < .05.

** *p* < .01. ^1^Statistic for the test of regression coefficients against 0.

## Discussion

The original English version of the CEBQ is a well validated and frequently used questionnaire which allows the assessment of children’s eating behaviour. Data analysis of the German and French questionnaire in this multi-lingual preschool study revealed a 7-factor structure instead of the original 8-factor structure of the CEBQ. The higher-order model including the two dimensions food approach and food avoidance as theoretically discussed and empirically previously investigated [15] was not supported using our data. Neither age nor gender of the child had any influence on the eating behaviour assessment, while for language area we found that French speaking parents reported that their children showed more FE and more SR than the parents of the German speaking preschool children, although these two effects were small and small to medium, respectively.

Like in our study, several studies using samples from other countries obtained a 7-factor structure [3, 18, 19, 22, 25] and the DD subscale was eliminated as it is suggested that a measurement model should consist of more than two items. Such a different factor structure solution has been repeatedly reported in many other studies, but some kept the original 8-factor solution because of a reasonable fit [16, 22] or to allow comparisons of their own results with other studies despite an unsatisfactory model fit [18, 23].

Only in one Portuguese study [21] did the authors remove the DD subscale like in our study, while the remaining studies with a different factor structure than the original version reported that other scales were excluded. One explanation for the difficulties of the DD subscale in our study might be, that the subscale DD does not explicitly assess the use of soft drinks [16], on which other studies had also revealed inconsistencies related to the concept of desiring to drink and the consumption of drinking sweetened beverages [30, 39] and young children as in our sample are likely to only rarely have access to soft drinks. Further, young children might have more difficulties to separate the feeling of hunger and thirst, and therefore consume more energy-dense beverages when feeling hungry [30] which might have impacted on our results in relation to the DD subscale.

Many studies refer to the two higher-order scales Food Approach and Food Avoidance [3, 15, 16, 18, 19, 21, 23]. The validity of these constructs could not be confirmed in our Swiss study. The sizes of both samples used by Ek [15] and the authors of the present study are comparable, although children in the Swedish study were slightly older (mean age 5.5) and included a small clinical sample (n = 47) with 20% of overweight children, which was not the case in the Swiss sample.

Further, there were a few adaptations needed in relation to the item distribution to the different factors in our statistical analyses. The item 3 (“My child has a big appetite”) was transferred in our study from SR to EF, as it loaded much higher on the latter. The difficulty with this item can be explained by the understanding of parents that a child with big appetite is rather considered as a positive aspect and rather related to enjoyment of food than satiety responsiveness at that age. This was also the case in a study with preschoolers [17] and further among infants less than 6 months old, where this item loaded comparably on the factor SR as on the three other factors EF, FF, and SE [8]. In other studies, similar problems were found with this item, but the loading was higher on subscale FR than on all the other subscales [19, 21].

Furthermore, two items in our study had to be removed to achieve a satisfactory model fit. Item 23 (“My child eats more when s/he is happy”), originally belonging to the EUE subscale (inversely coded) and item 28 (“Even if my child is full up, s/he finds room to eat his/her favorite food”) originally belonging to the FR subscale. Both loaded on several subscales and could not be assigned unequivocally. The difficulties to assess emotional eating might partly be explained by the fact that young children do not use eating as a coping strategy to emotional conditions yet [18], and that emotional eating is still less defined at this age period [16] and rather learned over the years. As we rely on parents’ reports of very young children where access to food is still limited, it remains unclear to which extent children might respond to emotional cues in case of free access to food at a later age period. We assume that parental assessment of eating behavior might have its own limitations as parents have few opportunities to compare their child’s eating behavior with that of others and they lack professional experience and expertise. This might influence their tendencies to respond to such questions. However, no such explanation can be used for low FR in relation to a favorite food, but other studies had revealed that FR and EOE might be overlapping concepts and therefore items might load on several subscales [18, 19].

It should also be kept in mind that apart from different study characteristics, the estimation methods used to analyse the statistical model may have an important influence on the results such as the estimates of the loadings or the goodness of model fit. As an example, in our study we considered the fact that the items underlying the latent constructs were ordinally scaled and that hence estimation methods such a maximum likelihood are not appropriate [40].

Analyses on the influence of sample characteristics revealed that French speaking preschool children showed more food enjoyment and a higher satiety responsiveness than children from the German speaking part of Switzerland, although the effect sizes were small to medium at best. Of note, we did not correct for multiple testing when reporting the estimates of the different loadings, therefore these two effects might represent chance findings. We therefore believe that our results are in line with other studies showing that the CEBQ is a reliable tool to be used in a multicultural context [16].

Further, our analyses revealed no impact of age or gender on any of the seven factors of the CEBQ although the sample covered a larger age range of 2- up to 6-year-old children and gender was almost equally distributed with 47% of girls and 53% of boys. The results are only partly in line with a study that focused on age and gender differences as well. Sleddens and colleagues [18] did not find any age difference either, but they compared only children aged 6 and aged 7 years. In contrast to our results, several studies had reported gender differences on factors of the CEBQ [18, 25, 27], but only one with a similar age group but in a small sample [18]. As our three explanatory variables explained only very little variance of any of the seven CEBQ factors, other factors might play a more important role in defining eating behaviour of a child such as parenting style [3, 41, 42], sedentary behaviour of relatives [43] and temperament characteristics of the child [31, 44, 45].

There are several limitations and strengths in this study. As strength, a sufficiently large community-based sample of children aged 2 to 6 years was assessed, although the number of children in the French speaking area was of somewhat limited size (n = 124). We applied suitable statistical methods to set up our 7-factor CFA model, taking into account the ordinal scaling of the items and we also reported the coefficient omega as a more useful measure of reliability of factors than Cronbach’s alpha. Also, in order to find out whether the factors of the CEBQ were influenced by gender, language area, or children’s age, used a MIMIC model, thereby taking into account the reliability of the different subscales of the CEBQ by including them as measurement models rather than computing sum scores which often leads to biased estimates [46]. Further, this is only the second empirical investigation to assess a possible higher-order factor model of the CEBQ, which our results could not corroborate. The limitations of the study are that we did not consider the differences in culture background which could influence the results. The CEBQ has been proven to be a reliable parental report to assess the child’s eating behaviour [14], but social desirability might still have influenced the response tendencies of parents. Further, as mainly mothers but also a few fathers have responded to these questions, gender differences of parents might have influenced the estimated child’s eating behaviour, but as the number of fathers responding to the questions still was low (14%). the impact of parental gender could not be considered in this study. Consequently, it might be preliminary to conclude on the test-theoretical quality of the current CEBQ and future studies should increase attempts to compare both parents’ view of the child’s eating behaviour. Furthermore, it is unclear whether eating behaviour is consistent over time, as children’s eating environment changes over time, it would be interesting in further studies to do a longitudinal analysis.

To sum up, this study aimed at validating the original factor structure of a French and German version of the CEBQ in a Swiss preschool sample and at identifying the impact of age, gender and language areas as sample characteristics on the observed factors in a large sample of preschool children including a broad age range. Our 7-factor version of the German and French CEBQ turned out to be both valid and reliable and might also be used in a multicultural context.

## References

[1] Ashcroft J, Semmler C, Carnell S, van Jaarsveld CHM, Wardle J. Continuity and stability of eating behaviour traits in children. Eur J Clin Nutr. 2008;62:985–90. doi: 10.1038/sj.ejcn.1602855 17684526

[2] Carnell S, Wardle J. Appetitive traits and child obesity: Measurement, origins and implications for intervention. Proc Nutr Soc. 2008;67:343–55. doi: 10.1017/S0029665108008641 18715519

[3] Jansen PW, Roza SJ, Jaddoe VWV, Mackenbach JD, Raat H, Hofman A, et al. Children’s eating behavior, feeding practices of parents and weight problems in early childhood: Results from the population-based Generation R Study. Int J Behav Nutr Phys Act. 2012;9(130):1–11. doi: 10.1186/1479-5868-9-130 23110748 PMC3543222

[4] Must A, Strauss RS. Risks and consequences of childhood and adolescent obesity. Int J Obes. 1999;23(Suppl 2):S2–S11. 10.1038/sj.ijo.0800852 10340798

[5] Randi G, Edefonti V, Ferraroni M, La Vecchia C, Decarli A. Dietary patterns and the risk of colorectal cancer and adenomas. Nutr Rev. 2010;68(7):389–408. doi: 10.1111/j.1753-4887.2010.00299.x 20591107

[6] Wang Y, Lobstein T. Worldwide trends in childhood overweight and obesity. Int J Pediatr Obes. 2006;1(1):11–25. doi: 10.1080/17477160600586747 17902211

[7] Powell F, Farrow C, Meyer C, Haycraft E. The stability and continuity of maternally reported and observed child eating behaviours and feeding practices across early childhood. Int J Environ Res Public Health. 2018;15(5):1–14. doi: 10.3390/ijerph15051017 29783638 PMC5982056

[8] Llewellyn CH, van Jaarsveld CHM, Johnson L, Carnell S, Wardle J. Development and factor structure of the Baby Eating Behaviour Questionnaire in the Gemini birth cohort. Appetite. 2011;57(2):388–96. doi: 10.1016/j.appet.2011.05.324 21672566

[9] Skinner JD, Carruth BR, Bounds W, Ziegler PJ. Children’s food preferences: A longitudinal analysis. J Am Diet Assoc. 2002;102(11):1638–47. doi: 10.1016/s0002-8223(02)90349-4 12449287

[10] Bilgin A, Baumann N, Jaekel J, Breeman LD, Bartmann P, Bäuml JG, et al. Early Crying, Sleeping, and Feeding Problems and Trajectories of Attention Problems From Childhood to Adulthood. Child Dev.2020; 91(1):e77–e91. doi: 10.1111/cdev.13155 30291757

[11] Barkley RA. Behavioral inhibition, sustained attention, and executive functions constructing a unifying theory of ADHD. Psychol Bull. 1997;121(1):65–94. doi: 10.1037/0033-2909.121.1.65 9000892

[12] American Psychiatric Association. Diagnostic and Statistical Manual of Mental Disorders (5^th^ edition). Arlington, VA: Author: 2013.

[13] Wardle J, Guthrie CA, Sanderson S, Rapoport L. Development of the Children’s Eating Behaviour Questionnaire. J Child Psychol Psychiatry. 2001;42(7):963–70. doi: 10.1111/1469-7610.00792 11693591

[14] Carnell S, Wardle J. Measuring behavioural susceptibility to obesity: Validation of the child eating behaviour questionnaire. Appetite. 2007;48:104–13. doi: 10.1016/j.appet.2006.07.075 16962207

[15] Ek A, Sorjonen K, Eli K, Lindberg L, Nyman J, Marcus C, et al. Associations between parental concerns about preschoolers’ weight and eating and parental feeding practices: Results from analyses of the child eating behavior questionnaire, the child feeding questionnaire, and the lifestyle behavior checklist. PLoS One. 2016;11(1). doi: 10.1371/journal.pone.0147257 26799397 PMC4723125

[16] Mallan KM, Liu WH, Mehta RJ, Daniels LA, Magarey A, Battistutta D. Maternal report of young children’s eating styles. Validation of the Children’s Eating Behaviour Questionnaire in three ethnically diverse Australian samples. Appetite. 2013;64:48–55. doi: 10.1016/j.appet.2013.01.003 23333562

[17] Quah PL, Fries LR, Chan MJ, Fogel A, McCrickerd K, Goh AT, et al. Validation of the Children’s Eating Behavior Questionnaire in 5 and 6 year-old children: The GUSTO Cohort Study. Front Psychol. 2019;10:1–9. 10.3389/fpsyg.2019.00824 31031683 PMC6470280

[18] Sleddens EFC, Kremers SPJ, Thijs C. The Children’s Eating Behaviour Questionnaire: Factorial validity and association with Body Mass Index in Dutch children aged 6–7. Int J Behav Nutr Phys Act. 2008;5(49). doi: 10.1186/1479-5868-5-49 18937832 PMC2612017

[19] Svensson V, Lundborg L, Cao Y, Nowicka P, Marcus C, Sobko T. Obesity related eating behaviour patterns in Swedish preschool children and association with age, gender, relative weight and parental weight—factorial validation of the Children’s Eating Behaviour Questionnaire. Int J Behav Nutr Phys Act. 2011;8:1–11. 10.1186/1479-5868-8-134 22152012 PMC3286377

[20] Webber L, Hill C, Saxton J, Van Jaarsveld CHM, Wardle J. Eating behaviour and weight in children. Int J Obes. 2009 Nov;33(1):21–8. doi: 10.1038/ijo.2008.219 19002146 PMC2817450

[21] Viana V, Sinde S, Saxton JC. Children’s Eating Behaviour Questionnaire: Associations with BMI in Portuguese children. Br J Nutr. 2008;100(2):445–50. doi: 10.1017/S0007114508894391 18275626

[22] Domoff SE, Miller AL, Kaciroti N, Lumeng JC. Validation of the Children’s Eating Behaviour Questionnaire in a low-income preschool-aged sample in the United States. Appetite. 2015;95:415–20. doi: 10.1016/j.appet.2015.08.002 26247701 PMC4589500

[23] Santos JL, Ho-Urriola JA, González A, Smalley S V., Domínguez-Vásquez P, Cataldo R, et al. Association between eating behavior scores and obesity in Chilean children. Nutr J. 2011;10(108):1–8. doi: 10.1186/1475-2891-10-108 21985269 PMC3213088

[24] Spahić R, Pranjić N. Children’s Eating Behaviour Questionnaire: Association with BMI in children aged 3–10 years from Bosnia and Herzegovina. Public Health Nutr. 2019;22(18):3360–7. doi: 10.1017/S1368980019002210 31391135 PMC10260618

[25] Cao Y-T, Svensson V, Marcus C, Zhang J, Zhang J-D, Sobko T. Eating behaviour patterns in Chinese children aged 12–18 months and association with relative weight—factorial validation of the Children’s Eating Behaviour Questionnaire. Int J Behav Nutr Phys Act [Internet]. 2012;9(5):1–7. doi: 10.1186/1479-5868-9-5 22272572 PMC3311563

[26] Lee MJ, Chang GT, Han YJ. Prestudy of validation of the Children’s Eating Behaviour Questionnaire in Korean children. Korean Orient Pediatr. 2009;23(1):127–40.

[27] Sirirassamee T, Hunchangsith P. Children’s eating behavior questionnaire: Factorial validation and differences in sex and educational level in Thai school-age children. Southeast Asian J Trop Med Public Health. 2016;47(6):1325–34. 29634198

[28] Quah PL, Cheung YB, Pang WW, Toh JY, Saw SM, Godfrey KM, et al. Validation of the Children’s Eating Behavior Questionnaire in 3 year old children of a multi-ethnic Asian population: The GUSTO cohort study. Appetite. 2017;113:100–5. doi: 10.1016/j.appet.2017.02.024 28232104 PMC5384631

[29] Blissett J, Haycraft E. Are parenting style and controlling feeding practices related? Appetite. 2008;50:477–85. doi: 10.1016/j.appet.2007.10.003 18023502

[30] Jalkanen H, Lindi V, Schwab U, Kiiskinen S, Venäläinen T, Karhunen L, et al. Eating behaviour is associated with eating frequency and food consumption in 6–8 year-old children: The Physical Activity and Nutrition in Children (PANIC) study. Appetite. 2017;114:28–37. doi: 10.1016/j.appet.2017.03.011 28315420

[31] Messerli-Bürgy N, Kakebeeke TH, Arhab A, Stülb K, Zysset AE, Leeger-Aschmann CS, et al. The Swiss Preschoolers’ health study (SPLASHY): Objectives and design of a prospective multi-site cohort study assessing psychological and physiological health in young children. BMC Pediatr. 2016;16(85):1–16. doi: 10.1186/s12887-016-0617-7 27390933 PMC4939002

[32] Ganzeboom HB. Occupational stratification measures for the new international standard classification of occupations (ISCO-08), with a discussion of the new classification. In: [Working Paper]. 2010.

[33] Hu LT, Bentler PM. Cutoff criteria for fit indexes in covariance structure analysis: Conventional criteria versus new alternatives. Struct Equ Model. 1999;6(1):1–55. 10.1080/10705519909540118

[34] Rosseel Y. lavaan: An R package for structural equation modeling and more. Version 0.5–12 (BETA). J Stat Softw. 2012;48(2):1–36. 10.18637/jss.v048.i02

[35] Woods CM. Evaluation of MIMIC-model methods for DIF testing with comparison to two-group analysis. Multivariate Behav Res. 2009;44(1):1–27. doi: 10.1080/00273170802620121 26795105

[36] Bollen KA. Issues in the comparative measurement of political democracy. Am Sociol Rev. 1980;45(3):370–90. 10.2307/2095172

[37] Trizano-Hermosilla I, Alvarado JM. Best alternatives to Cronbach’s alpha reliability in realistic conditions: Congeneric and asymmetrical measurements. Front Psychol. 2016;7:1–8. https://doi/10.3389/fpsyg.2016.00769 27303333 10.3389/fpsyg.2016.00769 PMC4880791

[38] Consortium PISA.ch. PISA 2018: Les élèves de Suisse en comparaison internationale [Internet]. 2019. Available from: https://www.pisa-schweiz.ch/eltern/publikationen/

[39] Sweetman C, Wardle J, Cooke L. Soft drinks and ‘desire to drink’ in preschoolers. Int J Behav Nutr Phys Act. 2008;5(60):3–6. doi: 10.1186/1479-5868-5-60 19055714 PMC2612018

[40] Brown TA. Confirmatory factor analysis for applied research. Guilford Publications. New York; 2006.

[41] Faith MS, Berkowitz RI, Stallings VA, Kerns J, Storey M, Stunkard AJ. Parental feeding attitudes and styles and child body mass index: Prospective analysis of a gene-environment interaction. Pediatrics. 2004;114(4):429–36. doi: 10.1542/peds.2003-1075-L 15466068

[42] Wake M, Nicholson JM, Hardy P, Smith K. Preschooler obesity and parenting styles of mothers and fathers: Australian National Population study. Pediatrics. 2007;120(6):1520–7. doi: 10.1542/peds.2006-3707 18055667

[43] Schmitz KH, Lytle LA, Phillips GA, Murray DM, Birnbaum AS, Kubik MY. Psychosocial correlates of physical activity and sedentary leisure habits in young adolescents: The teens eating for energy and nutrition at school study. Prev Med (Baltim). 2002;34(2):266–78. doi: 10.1006/pmed.2001.0982 11817924

[44] Haycraft E, Farrow C, Meyer C, Powell F, Blissett J. Relationships between temperament and eating behaviours in young children. Appetite. 2011;56(3):689–92. doi: 10.1016/j.appet.2011.02.005 21316412

[45] Tate AD, Trofholz A, Rudasill KM, Neumark-Sztainer D, Berge JM. Does child temperament modify the overweight risk associated with parent feeding behaviors and child eating behaviors?: An exploratory study. Appetite. 2016;101:178–83. doi: 10.1016/j.appet.2016.02.026 26916725 PMC4837692

[46] Newsom JT. Longitudinal structural equation modeling: A comprehensive introduction. Routledge. New York: Routledge; 2015.

